# Comparative transcriptomic analysis of surf clams (*Paphia undulate*) infected with two strains of *Vibrio spp.* reveals the identity of key immune genes involved in host defense

**DOI:** 10.1186/s12864-019-6351-4

**Published:** 2019-12-17

**Authors:** Mingjia Yu, Lin Zheng, Xiaobo Wang, Minfu Wu, Ming Qi, Wandong Fu, Yang Zhang

**Affiliations:** 1 Department of Food Science, Foshan Polytechnic, Foshan, 528137 China; 2 Zhejiang Marine Development Research Institute, Zhoushan, 316100 People’s Republic of China; 3 CAS Key Laboratory of Tropical Marine Bio-resources and Ecology, Guangdong Provincial Key Laboratory of Applied Marine Biology, South China Sea Institute of Oceanology, Chinese Academy of Science, 164 West Xingang Road, Guangzhou, 510301 China; 4 Innovation Academy of South China Sea Ecology and Environmental Engineering (ISEE), Chinese Academy of Sciences, Beijing, 100864 China

**Keywords:** RNA-seq, *Paphia undulate*, *Vibrio alginolyticus*, *Vibrio parahaemolyticus*, Virulence

## Abstract

**Background:**

*Vibrio spp.* is the major infection-producing marine bacteria in commercially important bivalve *Paphia undulata.* The host resistance is the major determining factor for the development of pathogenesis. To explore defense mechanisms, researchers have focused primarily on the study of differential expression of individual or specific groups of host immune genes during pathogen-challenge.

**Results:**

We compared the expression profile in the surf clams infected with avirulent *V. alginolyticus* and virulent *V. parahaemolyticus* to mark the possible molecular mechanisms of pathogenesis. Comparison of the differentially expressed genes between the two groups of *Vibrio-*infected clams revealed that the number of down-regulate genes in *V. parahaemolyticus* injected clams (1433) were significantly higher than the other group (169). Based on Gene Ontology classification, a large proportion of these down-regulate genes were found to be associated with cellular and molecular mechanisms for pathogen recognition, and immunity development thereby explaining the low survival rate for the *V. parahaemolyticus*-treated clams and suggesting a higher virulence of this bacterium towards the surf clams. Quantitative real-time PCR of 24 candidate genes related to immunity involving the JAK-STAT signaling pathway, complementary cascade, cytokine signaling pathway, oxidative stress, phagocytosis and apoptosis down regulated under *V. parahaemolyticus* infection, indicating compromised host defense*.* Furthermore, we could demonstrate a central role of JAK-STAT pathway in bacterial clearance. dsRNA mediated depletion of a clam STAT homolog gene results in dramatic increase in the infection by *V. alginolyticus*, a mildly pathogenic strain under control conditions.

**Conclusions:**

The difference in gene expression profiles in surf clams treated with two *Vibrio* species with a differential pathogenicity to *P. undulate* and downstream molecular analysis could enlighten on the probable molecular mechanisms of the *Vibrio* pathogenesis and the virulence of *V. parahaemolyticus* in surf clams, which also benefits to develop new strategies for disease control in surf calm aquaculture.

## Background

Bivalves are one of the earliest yet ubiquitous group of aquatic invertebrates with an estimated 10,000–20,000 living species. They are both economically and ecologically important with respect to food source, biomass and effects on communities. However, there is a steady progressive decline in the production of the bivalves following mass mortality among the farmed species due to marine microbial infections [1]. Till date, various species of the bacteria *Vibrio* and the protozoa *Perkinsus* have been identified as the major disease-producing pathogens affecting the development and survival of clams and diminishing the meat quality and thereby the price of the products [2]. The sedentary and filter-feeding habits among the bivalve mollusks lead to the accumulation of microorganisms (bacteria, fungi and parasites). These microorganisms besides being the source of nourishment also lead to the development of immune challenge in the mollusks [3].

The host resistance is the major determining factor for the development of pathogenesis. The defense mechanism in mollusks mainly relies on the effectors of innate immunity, which is mediated by circulating competent cells- referred to as hemocytes, and highly diversified humoral antimicrobial factors. Both these cellular and humoral components work in a synergistic way to initiate the recognition, segregation and ultimately elimination of pathogens and other non-self entities [4, 5]. The cellular response of innate immunity consists of three principle steps: (1) identification of pathogen-associated molecular patterns [PAMPs] by pattern recognition receptors [PRRs]; (2) activation of the regulatory pathways and (3) production of immune effectors to modulate cellular phagocytosis and to produce molecular effectors like antimicrobial peptides [AMPs] [6, 7]. In clams, phagocytosis and cytotoxicity are the two mechanisms for this cellular immunity; the latter involving the release of lysozymes, anti-microbial peptides, superoxide anion and hydrogen peroxide. On the other hand, humoral components include the lectin in addition to lysozymes and anti-microbial peptides [8]. Besides immunity, the hemocytes have various known functions including digestion, transport of nutrients, formation and mending of the shell, repair of wounds, excretion and internal defense [9]. Therefore, the molecular mechanisms for defense and other cellular and metabolic processes occurring in the hemocytes of clams during pathogen invasion are investigated to understand the host-pathogen interaction with a view to design therapeutic targets.

To explore defense mechanisms, researchers hitherto have focused primarily on the study of differential expression of individual or specific groups of host immune genes during pathogen-challenge. Recent application of high-throughput next generation sequencing technologies involving direct sequencing of transcripts (RNA-seq) are providing extensive information about host-microbe interactions at the transcriptional level including global gene expression and novel gene discovery [10–12]. The Solexa/Illumina and 454/Roche NGS technologies have been revolutionary for understanding the rich transcriptomes of the mollusks [13]. Due to its relatively low cost and good results obtained in different organisms, the Illumina RNA-Seq technology paired-end is a promising tool to study the clam immune system as well [11, 14, 15].

The surf clams are the bivalves supporting the largest proportion of the shellfishery market in China. In spite of it economic importance, the underlying molecular mechanism of surf clam defense towards *Vibrio*-infections remains largely unexplored. There are only two previous studies on the expression analyses of defense-related genes in surf clams (*Mesodesma donacium*) during *Vibrio spp*. (*V. anguillarum*)-challenge [16, 17]. In order to elucidate the immune mechanism associated with *Vibrio*-infection in surf calms, we utilized Illumina RNA-seq to score gene expression changes in *P. undulate* infected with two *Vibrio* pathogens- *V. parahaemolyticus* and *V. alginolyticus.* Of these two strains, *V. parahaemolyticus* was found to be more virulent than *V. alginolyticus,* as evidenced by the survival rate of *P. undulate* post pathogenic injection*.* Thus the comparison of the transcriptome of *P. undulate* infected with these two *Vibrio* strains could help us identify specific immune genes contributing to host resistance and molecular mechanism underlying the pathogenesis of marine molusks.

## Results

### *V. parahaemolyticus* is pathogenic towards *P. undulata*

**Fig. 1:**
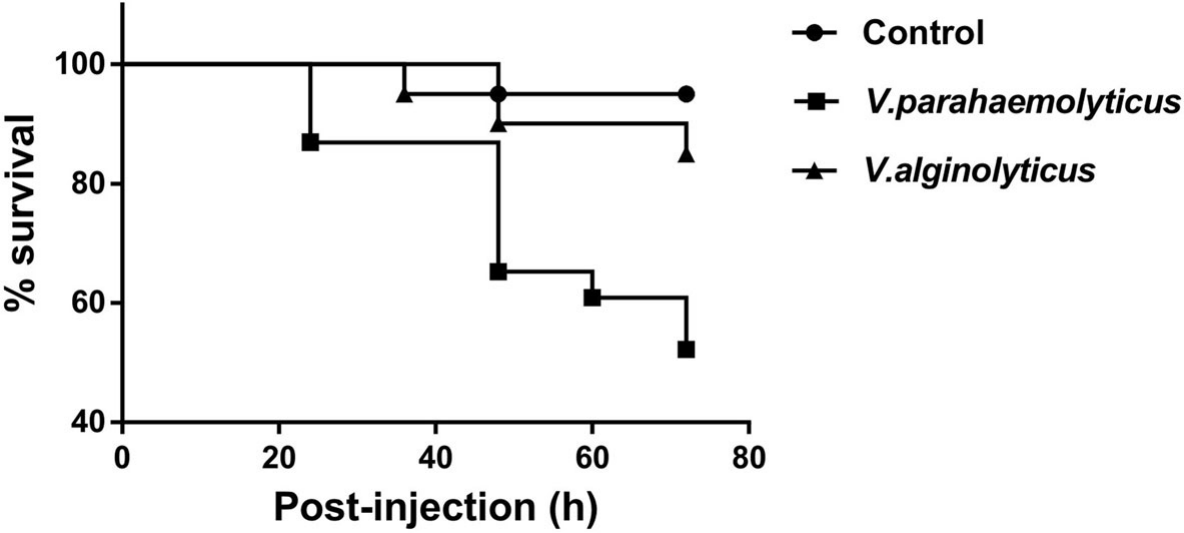
Comparison of rate of survival of surf clams treated with *V*. *alginolyticus* and *V. parahaemolyticus* with the controls (treated with PBS) from 24 h to 72 h post-challenge

To test the pathogenicity of the two *Vibrio* species, *V. parahaemolyticus* and *V. alginolyticus* towards surf clam *Paphia undulate*, the survival rate of the infected clams were measures at 24 h, 36 h, 48 h, 60 h and 72 h post-injection. A clear difference in the survivality was observed between clams infected with *V. parahaemolyticus* (VP) and the ones infected with *V. alginolyticus* (VA) in comparison to the controls (C) (Fig. 1). The survival rate of VA group was mostly comparable to the uninfected control group, C till 48 h post infection. At 72 h post-infection only a moderate decrease to 84.6% survival was noted in VA. In contrast, among VP group, the rate of survival of clams indicated a steep decline at 24 h (87.2%) and 48 h (65.3%) post-infection. At 72 h post-infection the percentage of surviving clams for VP decreased to 52.6%; thereby indicating a higher pathogenicity of *V. parahaemolyticus* towards surf clams.

### Transcriptomic analysis of *Vibrio* infected surf clams, *P. undulata*

**Fig. 2:**
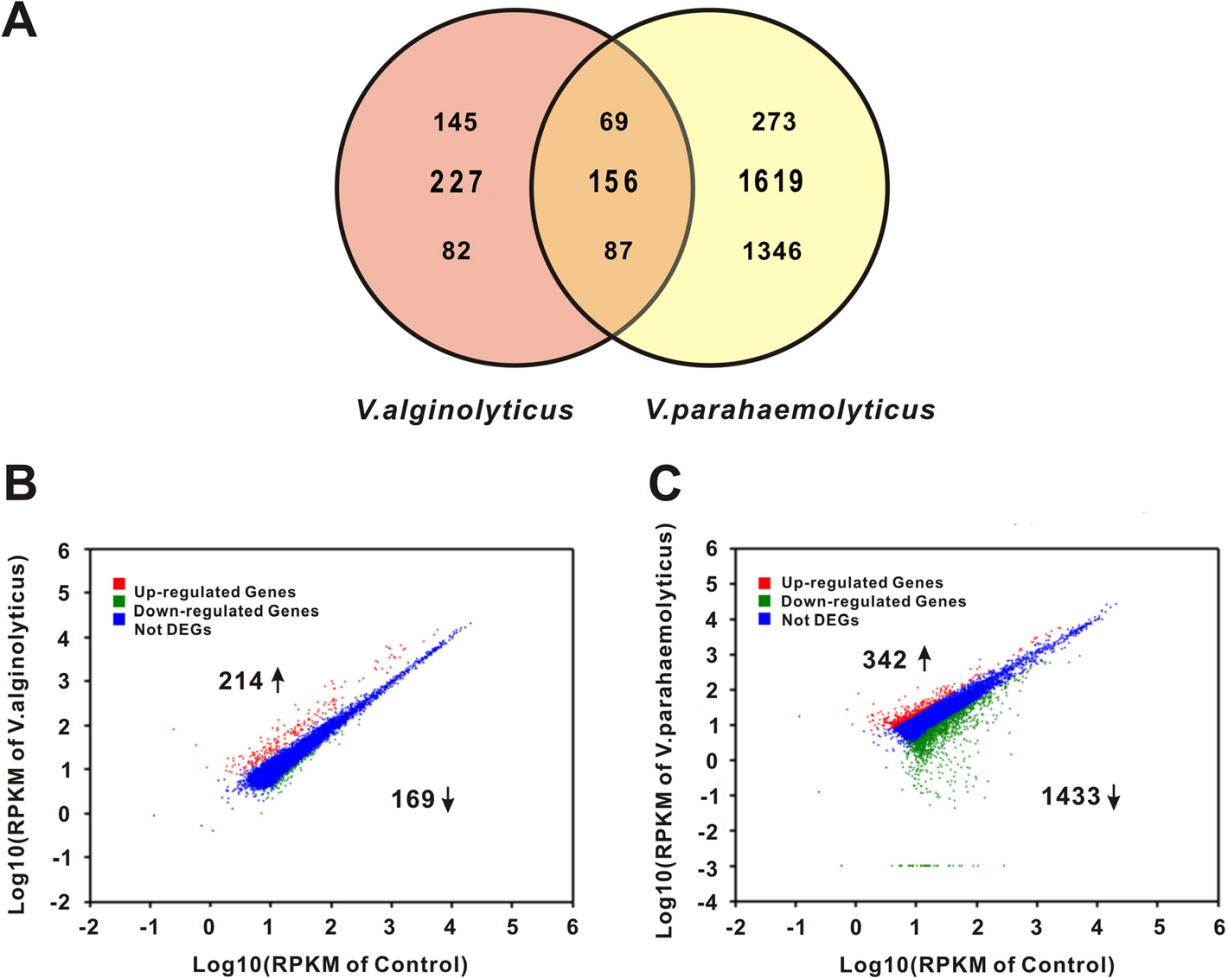
Comparative distribution of differentially and non-differentially expressed genes (DEGs and non-DEGs) in the surf clams treated with *V*. *alginolyticus* and *V. parahaemolyticus*. **a** Venn diagram representing the number of both common and exclusive DEGs and non-DEGs between the surf clams treated with *V*. *alginolyticus* and *V. parahaemolyticus* respectively. The numericals in the upper row, middle row and the lower row represent the numbers of up-regulated, non-DEGs and down-regulated genes respectively. **b** & **c** Normalized distribution of DEGs and non-DEGs obtained from *V*. *alginolyticus* (**b**) and *V. parahaemolyticus* (**c**) infected surf clam libraries

To gain better insight into mechanism of *Vibrio* mediated infection of surf clam *P. undulate*, high-throughput RNA-seq based transcriptomic analysis was performed. cDNA libraries were prepared for the *V. parahaemolyticus* and *V. alginolyticus* infected clams (VP and VA, respectively) and were sequenced using Illumina platform. All three libraries were assembled into annotated 74,433 sequences, which were used for references sequence for quantification analysis. The total mapped reads were 14,651,562, 13,544,017 and 14,529,523 for VP, VA and C groups respectively (Table 1). The percentage of clean reads in each library ranged from 52.04 to 55.11% of the total reads. The read summary of the sequences are provided in Table 1. Based on false discovery rate (FDR) ≤ 0.001, 766 and 3550 candidates were obtained from the VA and VP libraries, respectively. Using a cut-off criterion of Log10 fold change ≥ or ≤ 1, 383 and 1775 DEG were identified for VA and VP, respectively. Interestingly, we observed that a striking 1346 transcripts were found to be exclusively down-regulated in the VP group (Fig. 2a). Compared to the VA group the number of exclusive genes down-regulated in VP was much higher. Only 156 DEGs were shared between the two genesets, of which 69 and 87 were up and down-regulated respectively (Fig. 2a). The scatter plots showing the distribution of up and down regulated genes in VA and VP are provided with represented as log of RPKM values. The distribution of up-regulated and down-regulated genes in VA and VP with respect to control (C) is given by normalizing to RPKM values in Fig. 2b and c respectively.

**Table 1:** Summary statistics of the transcriptome assembled

Sample ID	Raw reads	Total base pairs	Total Mapped Reads	Perfect Match	<=2 bp Mismatch	Unique Match	Multi-position Match	Total Unmapped Reads
C	27921778	3490222250	14529523	9978734	4550789	13087865	1441658	13392255
VA	25931856	3241482000	13544017	9266974	4277043	12068700	1475317	12387839
VP	26587838	3323479750	14651562	10114803	4536759	13205013	1446549	11936276

### Functional analysis of genes affected by *Vibrio* infection

**Fig. 3:**
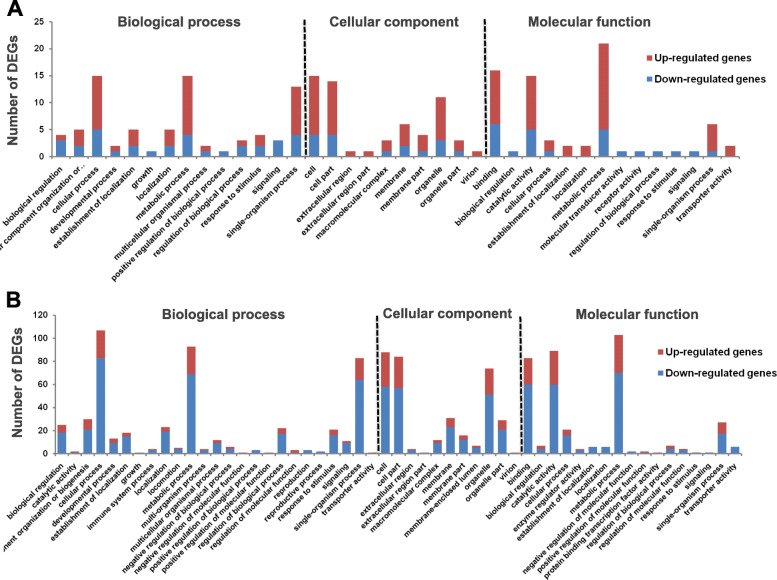
Summary of Gene Ontology (GO) functional annotation of DEGs in terms of biological processes, cellular components and molecular functions from *V*. *alginolyticus* (**a**) and *V. parahaemolyticus* (**b**) infected surf clam libraries

In order to get a better understanding about the *Vibrio* infection mechanism, a functional analysis of the DEGs were performed. Gene Ontology (GO) analysis showed that the DEGs were clustered into distinct groups (Fig. 3a and b). Of the 383 for VA and 1619 for VP had a GO ID and could be categorized into 55 functional groups. Strikingly, the most difference was that in contrast to large numbers of mapped up-regulated genes in VA and most of the mapped VP genes were down-regulated (Fig. 3b). For biological process category, the most abundant genes were identified for cellular process (110 DEGs for VP), metabolic process (90 DEGs for VP) and single-organism process (80 DEGs for VP). For cellular component category, the most abundant genes were identified for cell (85 DEGs for VP), cell parts (82 DEGs for VP) and organelle (75 DEGs for VP). For molecular function category, the most abundant genes were identified for binding (75 DEGs for VP), catalytic activity (84 DEGs for VP) and metabolic processes (98 DEGs for VP). Similar functional categories were also found to be significantly effected in VA geneset as well. Additionally, detailed analysis revealed that transcript assignment to GO terms identified genes related to pathogen recognition, binding and innate immunity of surf clams which were all down regulated in VP but were either up-regulated or did not show any variation in expression in VA. These include immune system process (2 DEGs); response to stimulus (18 DEGs); macromolecular complex (15 DEGs); membrane (30 DEGs) and membrane part (18 DEGs). All the genes involved in establishment of localization (3DEGs for VA and 10 for VP) and localization (3DEGs for VA and 10 for VP) were found to be up-regulated in VA and down-regulated in VP libraries. In summary, these terms account for a large fraction of the overall assignments in the transcriptome of the surf clam (Table 2).

**Table 2:** Classification of the transcriptome according to GO terms

GO accession	GO term	Functional category	No of DEGS	
			VP	VA
GO:0009987	Cellular process	Biological process	110	15
GO:0008152	Metabolic process		90	15
GO:0022413	Single organism process		80	13
GO:0002376	Immune system process		2	0
GO:0005623	Cell	Cellular component	85	15
GO:0044464	Cell parts		82	13
GO:0043226	Organelle		75	12
GO:0032991	Macromolecular complex		15	3
GO:0016020	Membrane		30	5
GO:0044425	Membrane part		18	3
GO:0005488	Binding	Molecular function	75	17
GO:0003824	Catalytic activity		84	16
GO:0008152	Metabolic process		98	20
GO:0050896	Response to stimulus		18	1
GO:0051234	Establishment of localization		10	3
GO:0051179	Localization		10	3
GO:0005215	Transporter activity		5	3
GO:0022413	Single organism process		20	7

### *V. parahaemolyticus* infection results in the suppression of key immune genes in the clam *P. undulata*

**Fig. 4:**
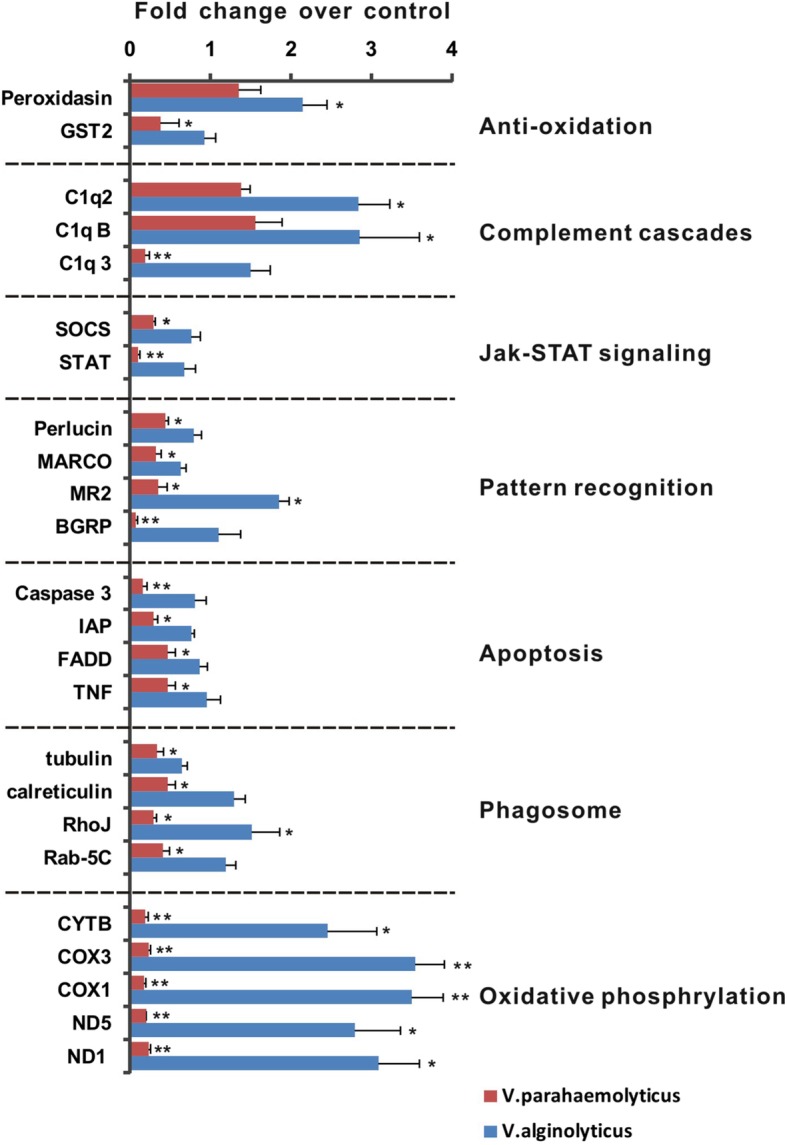
Validation of expression profile of the DEGs obtained from *V*. *alginolyticus* and *V. parahaemolyticus* infected surf clam libraries by quantitative real time PCR (qRT-PCR). Control = Expression of beta-actin gene from the infected surf clams. Bars with asterisks indicated values that were significantly different (*P* < 0.05) from control samples. Error bars indicated standard deviations of averages from 5 replicates

**Fig. 5:**
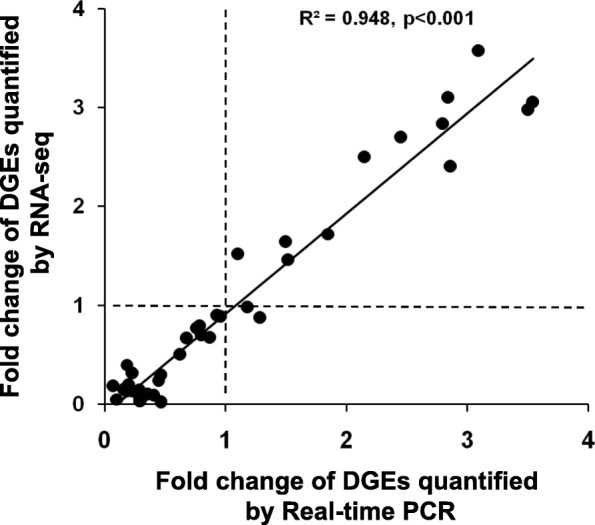
Scattered plot analysis of comparing expression correlation of candidate DEGs through RNA-sequencing on Illumina platform and qRT-PCR. Each point represents one DEG with paired expression value. X-axis and Y-axis indicates the expression value that quantified by Real-time PCR and RNA-seq, respectively

Repression of large number of immune genes in *P.undulata* haemocytes infected with *V. parahaemolyticus* led us to undertake a through qRT-PCR based analysis of candidate immune genes. Twenty four innate immunity or immunity related genes involved in anti-oxidation, complement cascade, JAK-STAT signaling, pattern recognition, apoptosis, phagosome and oxidative phosphorylation were selected for the analysis (Table 3). Strikingly, all the 24 gene assayed showed a reduced expression in the VP group as compared to the VA, and the repression was particularly prominent for C1q3 of complement cascade, STAT of JAK-STAT signaling pathway, MR2 and BGRP pattern recognition proteins, Caspase 3 of apoptotic pathway and Rho-J and Rab-5C involved in phagosome formation. Additionally, all the tested members of oxidative phosphorylation- CYTB, COX3, COX1, ND5 and ND1 were drastically repressed in VP (Fig. 4). Additionally, these qRT-PCR analyses validate our Illumina RNA-seq results to a large extent. The correlation of the fold change of DEGs obtained by Illumina RNA-seq and qRT-PCR was analyzed by scattered plot (Fig. 5). The pattern of fold change of DEGs observed from Illuminia RNA-seq well corroborated with that from qRT-PCR (R^2^ = 0.948 and *p* value< 0.001). The values were mostly clustered between 0.5 and 1 while very few remain scattered between the ranges 2 to 4 for both the dataset. Therefore, qRT-PCR data supported the sequencing results and provided data about the suitability of using the Illumina sequencing approach for de novo assembly of the surf clam hemocytes transcriptome without a reference genome.

**Table 3:** Sequences of the primers used in this study

Gene Id	Protein	Function	Primer sequence
Peroxidasin	Peroxidase	antimicrobial defense	F: TAGAAACAGCGTCCTCAACAGTTAG R: ATCATAATCCGGTGTTAAAG
GST2	Glutathione S-transferase	detoxification (oxidative stress reponse)	F: TTCATCTTCACCTTCCGAACTAAAG R: TGCCAGTGCTTGAAATAACCGAC
C1q2	Complement like factors	Pathogen recognition	F: TTAGCAATATCATACGGGATAG R: AGAACAGCTGTAAATGCGATGACAT
C1qB	Complement like factors	Pathogen recognition	F: TTAATACAAATGTTGTTGCCGACAC R: TGTGGCCATTGAATGCTTATTGC
C1q3	Complement like factors	Pathogen recognition	F: GCGTTTTGGTGACAATTACATGTTC R: TTGGTCAAATTTTATTACTAAGCC
SOCS	Suppressor of cytokine signaling	signal transduction	F: AAACCGACGGTAACGAGAAT R: TAAATATTTAGAATCCGAACTATCA
STAT	Signal transducers and activators of transcription	signal transduction	F: ATCCCGTATTTCTGCTCGGC R: GTGCGATGGCTTGTTCATGG
Perlucin	Shell matrix protein/defense molecule	cellular component/ immunity	F: TCTACGTTTGGCTGAAGTCGGTCTA R: GATGGCCCTTATATGTCAAT
MR2	Macrophage receptor with collagenous structure	Scavenging (innate immunity)	F: CAGGCAAGTGTTTTCTCGTGTTGGC R: GTCCGGCAAGGTAGTAGCTT
BGRP	Beta-glucan recognition protein	Pattern recognition (innate immunity)	F: AACGGCATATCTTTAGTAGCAT R: TGAGGTTGTTGGACTAGACGCTGTC
Caspase 3	Protease	Apoptosis	F: CCTCCAGAACCAAGAAGCGT R: CTGGGGTTAAGATGCCACGT
IAP	inhibitor of apoptosis	balance between cell proliferation and cell death by inhibiting caspase activity and facilitating immune responses	F: TATGGTAAAATGGAAGACGC R: CCACCACTGCTTCTTTGTCTAAACA
FADD	Fas-Associated protein with Death Domain	apoptosis	F: GGTACACCAAGCTCTCGCAT R: TGAGAGGACATGTCGAGGCT
TNF	Tumor necrosis factor	immunity	F: TGGTTGTTCTGCATTCGCTTGTTAC R: AATGTTCAGAAATCGGAATTGGT
Tubulin	centrosomal protein	Cell division; oxidative stress	F: AGAGACTGGAGCTGGCAAACACGTA R: GAGCATAGTTGTTGGCAGCGTC
Calreticulin	Endoplasmic reticulum chaperon	Calcium homeostasis and protein maturation; oxidative stress	F: AGATATGTACGGAGAATCACCTTAC R: TACCACTTTCTACTTTAGCGTT
Rho-J	GTP-binding protein	Signal transduction	F: GAAGGACTGCGCGTGTTTACTTAC R: TTGCTCCAATCTTATTCGCCAATTT
Rab-5C	Ras-related protein	Endocytosis	F: GCCGACTGAGGTCTTAACTT R: ACAAGCAGCCGTCGTAGTATATGAC
CYTB	cytochrome b oxidase	Oxidative phosphorylation	F: ACAAGACTCCGGCGCATATT R: AAAGGTCTTTCCACAGGGCAAGTCC
COX3	cytochrome c oxidase subunit 3	Oxidative phosphorylation	F: CTGCAGTATTCGGAGTATAAGTGGT R: AACGACGGAATGCGAAGTGAT
COX1	cytochrome c oxidase subunit 1	Oxidative phosphorylation	F: GTTACTGCTCATGGGCTAGTG R: ATCCTCAACCCAAACAGACCTTAAT
ND5	NADH dehydrogenase subunit 5	Oxidative phosphorylation	F: GGGGGTATATGTATTACTTC R: CTCCCAATCAAAAACACTATAATCC
ND1	NADH dehydrogenase subunit 1	Oxidative phosphorylation	F: CCCCGCCCCGTATTCTACAT R: ATAAGGGTTATTATTTGGGCAGGC

### JAK-STAT pathway plays a key role in bacterial clearance

**Fig. 6:**
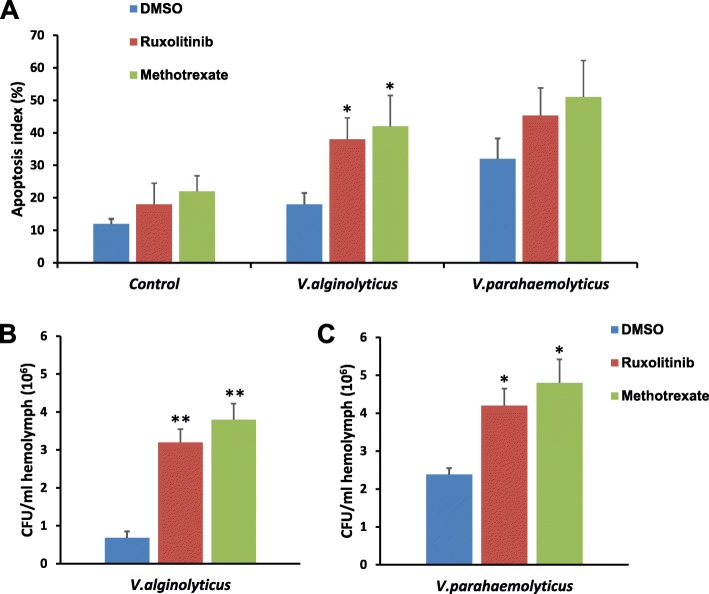
JAK-STAT pathway plays a key role in bacterial clearance. **a** & **b** Bacterial count (Colony forming units, CFU) in *V.alginolyticus* (**a**) and *V.parahemolyticus* (**b**) infected surf clams treated with JAK/STAT pathway inhibitors Ruxolitinib and Methotrexate. DMSO treatment was used as controls. *N* = 3. **c** Apoptosis index (%) in *V.alginolyticus* and *V.parahemolyticus* infected surf clams treated with JAK/STAT pathway inhibitors Ruxolitinib and Methotrexate. DMSO treatment was used as controls. The infected clams were compared with uninfected controls. *N* = 5

To further investigate the involvement of JAK-STAT pathway in successful establishment of *Vibrio* infection in surf clams, we utilized JAK-STAT pathway inhibitors- methotrexate and ruxolitinib. We investigated infection induced apoptosis in JAK/STAT pathway inhibitor treated surf clams. Suppression of JAK/STAT pathway induced apoptosis in VA infected clams, and the observed apoptotic index was significantly higher than DMSO treated controls (Fig. 6a). Inhibitor treatment did not induce apoptosis in uninfected controls, indicating that the observed increase is due to the successful establishment of VA infection (Fig. 6a). VP, on the other hand could induce apoptosis in DMSO treated controls, which was moderately enhanced under JAK/STAT pathway inhibitor treatment (Fig. 6a). The apoptotic index of VA and VP infected inhibitor treated clams were mostly comparable (Fig. 6a). Additionally, we also monitored the effect of JAK/STAT pathway on ability of bacterial clearance in surf clams. As expected, the bacterial count in VA infected and control (DMSO treated) clams were low in comparison to VP infected ones presumably due to the lower virulence of VA. Interestingly, treatment with either methotrexate or ruxolithinib of the clams leads to a significant increase in bacterial count under VA infection (Fig. 6b and c). Since VA is mildly virulent to *P. undulate*, the above results indicate a central role of JAK/STAT pathway against *Vibrio* defense. Moderate changes in the bacterial count were also noted in VP infected, inhibitor treated clams. Therefore, considering all these evidences, it can be concluded that in surf clam *P. undulate* JAK-STAT pathway plays a crucial role in bacterial clearance; inhibition of which results in establishment of successful infections in clams by a virulent VA strains as well.

### Identification of putative STAT gene involved in clam defense

**Fig. 7:**
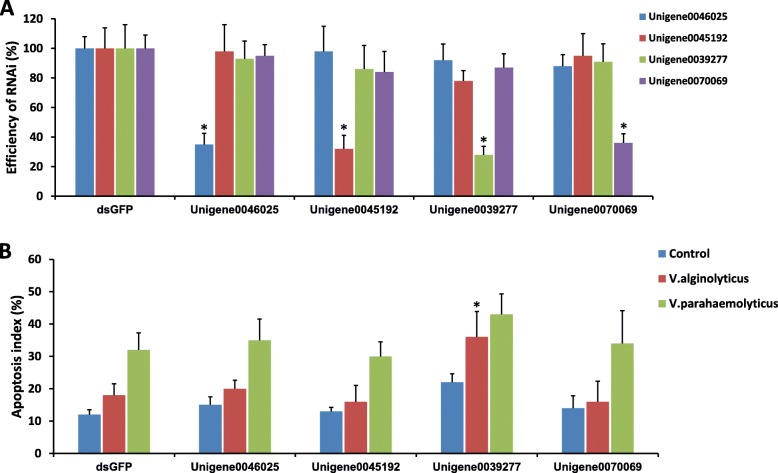
The effect of dsRNA mediated knockdowns of putative clam STAT genes on Vibrio infection as evidenced by measuring the infection-induced apoptosis index. **a** The efficiency and specificity of dsRNA knockdowns were verified by the Realtime PCR analysis. *N* = 5. **b** Two Vibrio infection induced apoptosis index (%) of hemocytes were assessed after knockdowns the STAT genes. Clams injected with dsRNA for GFP were used as controls. *N* = 5

Based on our transcriptome mapping, four putative genes- unigene0046025, unigene0045192, unigene0039277 and unigene0070069, were annotated to the STAT protein in *P. undulate*. To elucidate their possible role in *Vibrio* infection, we performed dsRNA mediated knockdown of the four genes mentioned above in surf clam. The efficiency and specificity of the target gene knockdowns were tested by qPCR (Fig. 7). Clams treated with double-stranded RNA against green fluorescent protein (GFP) were used as controls. Putative STAT gene depleted clams were then infected with either *V. alginolyticus* or *V. parahymolyticus* (Fig. 7a). To access infection levels, apoptosis index were measured under various treatment conditions. In the control (dsGFP), VA infection results in an apoptosis index similar to uninfected calms, while a significantly higher apoptosis was observed under VP infection reflecting on the higher virulence of VP, mentioned above (Fig. 7b). Interestingly, knockdown of unigene0039277 results in a significant increase in the VA infection apoptosis index, which is noticeably higher than uninfected controls. This result suggests a key role of the clam unigene0039277 in combating *Vibrio* infection, the loss of which results in higher virulence of mostly non-pathogenic VA. A significant but lesser increase in the apoptotic index was observed under VP infection in unigene0039277 depleted clams as well (Fig. 7b). Additionally, unigene0039277 being mapped to clam STAT protein, the above experiment strongly support our previous results on the central role of JAK/STAT pathway in clam defense against bacterial infection. However, knockdown of other unigenes mapped to STAT does not play a noteworthy role against *Vibrio* attack, as evidenced by the similarity in apoptosis index derived infection profile with the control.

### STAT transcriptionally regulates the expression of other immune genes in *P. undulate*

**Fig. 8:**
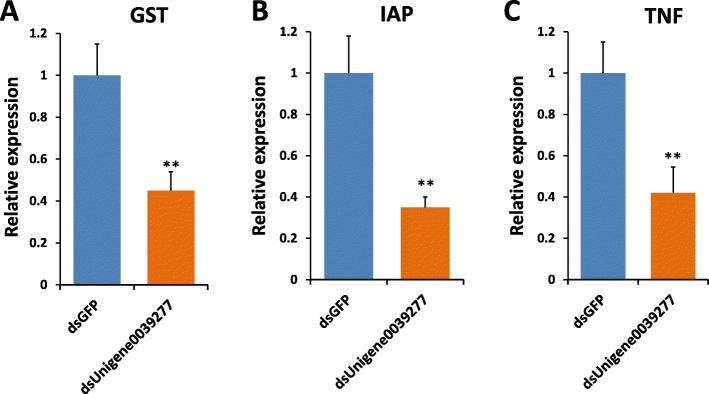
The effect of knockdown STAT gene homolog, unigene0039277, on the expression of several candidates genes involved in clam defense. A clear reduction in the expression levels of (**a**) glutathione-S-transferase (GST), (**b**) inhibitor of apoptosis (IAP) and (**c**) tumor necrosis factor (TNF) was observed in dsUnigene0039277 clams. *N* = 5

To further investigate the role of unigene0039277, we tested the expression of key defense related genes- glutathione-S-transferase (GST), inhibitor of apoptosis (IAP) and tumor necrosis factor (TNF) in unigene0039277 depleted clams. GSTs are proteins which help in cellular detoxification by conjugating the toxins with glutathione (GSH), increase their solubility and thereby aiding to the easy removal of the toxins from the cells [20, 21]. On the other hand, IAPs are the class of proteins that suppress host cell death by inhibiting caspases during infection [22]. In addition, IAP is related to signal transduction pathways used by TNF-receptors. This TNF plays role in systemic inflammation and is a cytokine involved during acute phase infection by regulating the immune cells [23]. TNF stimulates phagocytosis and promotes the expression of adhesion molecules on endothelial cells; thereby helping in the migration of neutrophils. Our qRT-PCR analysis revealed that the basal expression of all the three genes tested were compromised in unigene0039277 depleted uninfected clams (Fig. 8). Specifically, the relative expression for GST, IAP and TNF was found to be 43.8, 32.5 and 41.2%, respectively of the dsGFP controls (normalized to be at 100% level) (Fig. 8). Thus JAK-STAT pathway, particularly unigene0039277, regulates the transcription of key immune genes in clams.

### STAT gene homolog imparts resistance against *Vibrio* attack

**Fig. 9:**
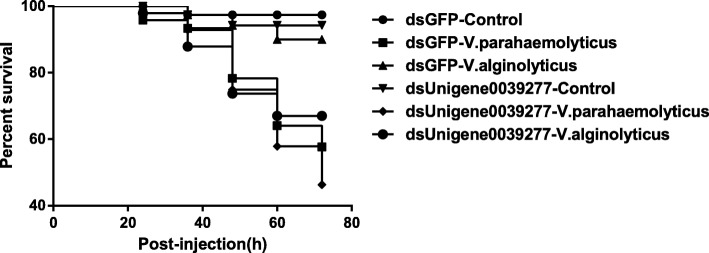
Survival rate of *Vibrio*-infected clams treated with dsRNA against the STAT homolog, unigene0039277. dsGFP injected clams were used as controls. Uninfected dsGFP and dsUnigene0039277 clams were used for the purpose of comparisons. Each treatment or control contained 18–20 individuals

Next, we investigated the effect of unigene0039277 on the survival of surf clams under VA and VP infection. dsGFP injected clams were used as controls and uninfected dsGFP clam showed a near 100% survival rate at about 70 h post infection (Fig. 9). The observed results for dsGFP are similar to that described previously for untreated clams (Fig. 1). Uninfected unigene0039277 depleted clams showed a survival rate slightly lower (96.6% at 72 h post injection) than dsGFP controls. However, under VA infection, prominent differences were observed in survival rates between dsGFP (91.7% at 72 h post injection) and unigene0039277 depleted (67.4% at 72 h post injection) groups, with a drop of nearly 24.3% (Fig. 9). Thus, it can be assumed that the loss of this key gene unigene0039277 transforms the mildly pathogenic VA strain to a virulent one. Loss of unigene0039277 also impacts the VP infection and the survival rate drops even further compared to VP infected dsGFP injected controls (Fig. 9). Taken together, our results suggest a key role for the STAT homolog unigene0039277 in resistance against *Vibrio* infection in *P. undulate*, and presumable the virulent strain *V. parahaemolyticus* establishes infection by suppressing this gene by utilizing a yet unknown mechanism. The suppression of this gene is presumably a key step in the establishment of the virulent strain *V. parahaemolyticus.*

## Discussion

Aiming to elucidate the mechanism of *Vibrio* infection in surf clams, RNA-seq based transcriptomic analysis was performed with the hemocyte from infected *P. undulate*. Two strains of *Vibrio* were used, one being less pathogenic than the other. While *V. parahaemolyticus* infection resulted in a dramatic reduction in the survival of surf clams, the *V. alginolyticus* infected clams could overcome the bacterial challenge and had a survival curve similar to the controls. This clearly indicated the differential pathogenicity of these two *Vibrio* strains, and established *V. parahaemolyticus* as a more virulent strain than *V. alginolyticus.* The comparison of these two transcriptomes and follow up qRT-PCR experiments revealed the identity of certain genes which might be involved in the process of pathogen establishment. Comparison of the two transcriptomes revealed that the expressions of a much higher number of genes were affected by *V. parahaemolyticus* infection in contrast to *V. alginolyticus,* in surf clam *P. undulate.* As a consequence, a significantly high number of DEGs from the *V. parahaemolyticus* infected surf clams was observed for all the three major functional categories of GO database- biological process, cellular components and molecular functions. Most of these genes were enriched in GO terms as “cellular process”, “metabolic process”, “single organism process”, “cell”, “cell parts”, “organelle”, “macromolecular complex”, “membrane”, “membrane part”, “binding”, “catalytic activity”, “metabolic process”, “response to stimulus”, “establishment of localization” and “localization”. A similar distribution of terms was observed for the *V. alginolyticus* infected clam DEGs mapped to GO database. In this regard, it can be mentioned here that, previous studies have generally identified similar GO slim terms associated with DEGs from different tissues of various bivalve species [9]. However, as compared to up-regulated ones, the number of down-regulated genes was significantly higher in the *V. parahaemolyticus* infected group of surf clams. This was in contrast to *V. alginolyticus* where the higher numbers of mapped DEGs were associated with the up-regulated geneset. The VP down-regulated genes included significant number of candidates involved in clam immunity or immune related function.

Innate immunity is the major component of invertebrate immune system that is triggered by the recognition of conserved PAMPs present in the microbes by PRRs in the host [24]. Common PRRs include toll-like receptors (TLR), retinoic acid-inducible gene I [RIG-I]-like receptors (RLRs) and NACHT-leucine-rich repeat receptor (NLR) [24]. Pathogen recognition is followed by the binding of the pathogen with the cell membrane and the subsequent chemotactic migration of the immune cells like hemocytes towards invading pathogens. PAMP-PRR interaction activates intracellular signaling pathways, including adaptor molecules, kinases, transcription factors, trigger proinflammatory and antimicrobial effectors. Finally, the hemocytes phagocytose and kill these invaders by producing lysozymes, AMPs and toxic radicals. Phagocytosis is one of the most important defensive functions of hemocytes during which the immune cells recognize and eliminate non-self-components including invading and/or associating microorganisms [25]. During phagocytosis, phagosomes interact with the parts of the cell membrane and with the organelles including recycling endosomes, late endosomes and lysosomes [9]. The phagosomes get fused to the lysosomes forming phagolysosomes and release toxic products like reactive oxygen species, nitrogen species and proteins such as defensins and endopeptidases and exopeptidases, hydrolases and proteases which kill the pathogen and degrade them into fragments [9]. Additionally, immediately after pathogenic attack, the complement cascade is also activated which aids host defense [26].

In our study, 24 candidate genes were selected for qRT-PCR analyses based on their implication in the host-immune response towards *Vibrio spp.* and their differential expression observed in the clam hemocyte transcriptomic library. Most of the immune genes tested were highly induced under VA infection, indicating development of strong immune response in the host. However, a clear repression of several tested genes was observed in case of VP infection in comparison to VA. This repression of host immune genes might help explain the enhanced pathogenicity observed for VP. Among them, the Janus kinase (JAK)- signal transducers and activators of transcription (STAT) pathway is a pleiotropic signal transduction cascade that transduce a multitude of signals critical for cellular homeostatis, development and immunity [27]. Mechanistically, the JAK-STAT pathway is relatively simple consisting of three principal components- a membrane receptor for the extracellular signal, a JAK tyrosine kinase and STAT proteins [28]. The signaling pathway is activated by the binding of a variety of ligands including cytokines and growth regulators to the transmembrane receptor [29]. As a result, the receptor associated JAK proteins are activated through a conformational change which in turn phosphorylates latent STAT monomers leading to its dimerization, nuclear localization and DNA binding [27, 30–32]. The effect of the pathway is finally manifested by alterations in the expression of JAK-STAT pathway target genes, providing a direct mechanism to translate an extracellular signal into a transcriptional response [29]. In addition the core components, the JAK-STAT pathway have other effector like adapters, pathway effectors and several pathway inhibitors (like suppressors of cytokine signaling (SOCS), protein inhibitors of activated STATs (PIAS), protein tyrosine phosphatases (PTPs) etc.). JAK-STAT is a classics signaling pathway that is evolutionarily conserved from vertebrates to lower organism inducing bivalves and other economically important marine organisms.

The JAK/STAT pathway is one of the major signaling cascades required for providing resistance against pathogenic attack, both in vertebrates and invertebrates. Although the pathway was initially discovered while researching antiviral mechanisms of interferons, it was later shown to play significant role against other pathogens as well [30]. Anti-viral role of JAK-STAT pathway has been widely reported in various organisms including *Drosophila*, mice and humans [30, 33]. Loss of function mutation of JAK-STAT pathway leads to impaired anti-fungal defense in fruit fly *Drosophila melanogaster* [34, 35]. Particularly interesting is the finding that JAK-STAT pathway can provide resistance against Dengue virus in the disease carrying vector mosquitoes [35]. Anti-bacterial role of JAK/STAT pathway has also been widely reported with various effector genes identified to be regulated by the pathway [36, 37]. JAK/STAT pathway has been consistently linked with antibacterial response in the gut in several organisms, where the pathway was demonstrated to induce production of anti-microbial peptides (AMPs) [37, 38]. There are relatively fewer studies reporting the role of JAK-STAT pathway in anti-fungal defense [38]; however several papers have demonstrated involvement of the pathway in resistance against the entomopathogenic fungus *Beauveria bassiana* in insects [38, 39]. Additionally, JAK-STAT pathway has also been implicated to play a role in encapsulation response in invertebrates, primarily via facilitating the differentiation of the hemocytes [40]. Encapsulation provides defense against parasites and foreign objects that invade the invertebrate circulatory system. The phenomenon is particularly prevalent amongst insects where JAK-STAT pathway is widely shown to induce encapsulation of parasitoid wasp eggs [40]. Thus, in additional to the multitude of physiological roles including development and homeostatic, JAK-STAT pathway plays a significant role in immune resistance against all kinds of pathogens and is a critical of component of host defense system.

In comparison to the wealth of information available for JAK-STAT pathway and its biological function in vertebrates and insects, relatively few studies have investigated the same in ecologically and economically important marine organisms. Although there are reports about the involvement of JAK-STAT pathway in anti-pathogen defense in various aquaculture organisms including shrimps and crabs [41, 42]. In mammals, four JAKs (JAK1, JAK2, JAK3, TYK2) and seven STATs (STAT1, STAT2, STAT3, STAT4, STAT5a, STAT5b, STAT6) have been reported [30]. In invertebrate species however, fewer homologs of JAK and STAT are usually reported, with most of the species harboring just one to two JAK and one STAT protein. Recent studies focus on JAK-STAT pathways in economically important mollusks including bivalves have identified the presence of JAK and STAT homologs as well as homologs of other important members of JAK-STAT pathway [18, 19]. Interestingly, like other organisms, a role of the pathway in anti-pathogenic defense has been reported in bivalves including mussel and oysters [19, 43].

In this study, JAK/SAT pathway plays a key role in bacterial clearance as evidenced by increased infection under inhibitor treatment in surf clams *P. undulate*. Knockdown of a particular putative STAT gene increased the virulence of *V. alginolyticus*, a bacterium generally perceived to be mildly pathogenic to *P. undulate.* Thus, we assume that VP, which is virulent to *P. undulate*, functions by suppressing the STAT homolog (unigene0039277). Interestingly, STAT3-dependent signal transduction has also been reported to be involved in immune response in whiteleg shrimp, *Litopenaeus vannamei* during *Vibrio spp.* infection. Increased mortality in *Vibrio*-infected *Litopenaeus vannamei* was reported due to inhibition of JAK/STAT pathway by cell death regulatory genes indicating that the latter was necessary for anti-bacterial defense [44]. However, the exact molecular mechanism by which JAK-STAT pathway provides resistance against *Vibrio* infection in surf clam remains to be elucidated. Interestingly, knockdown of the STAT homolog, unigene0039277, in clams resulted in repression of several other key immune genes like GST, IAP and TNF inspiring the idea that surf clam STAT indeed might be a key gene that regulate other immune genes in a systematic fashion. A thorough analysis of unigene0039277 knockdown clam is required to address these questions in the future.

## Conclusions

The ability of a host organism to overcome a pathogenic attack depends on how successfully it can mount a defense in response to pathogenic attack. On the other hand, a pathogenic establishment requires overcoming or bypassing this host defense [45]. Pathogenic contact often results in induction in the expression of several key immune genes [45]. This induction of host immune genes has been found to be compromised in case of virulent pathogenic attack, which finally overcomes host defense to establish itself [45]. In our study, we observe the induction of several immunity related genes (as discussed above) in clams infected with *V. alginolyticus.* The expression of some of these genes remained low at a level almost comparable to the controls, in case of *V. parahaemolyticus* infection.

To the best of our knowledge, this is the first study on the investigation of transcriptome profile of the surf clams during the challenge by virulent *Vibrio* strain. The variation of expression of a large number of genes detected by RNA sequencing in our study provides a rich resource for studying the novel genes involved during *Vibrio*-challenge in surf clams. Comparative genomic analysis of *P. undulate* infected with two different *Vibrio* strains helped us demostrate key role of JAK/STAT pathway in clam defense and pathogen clearance. A specific putative STAT gene (unigene0039277) was found to provide enhanched resistance against *Vibrio* attack, and the suppression of this gene is presumed to be an important step in the establishment of infection by the virulent strain *V. parahaemolyticus.* However, the exact mechanism of repression of unigene0039277 or other immune genes remains unclear. Our findings might lead to the identification of therapeutic targets for infection minimization further helping us maintain ecological and economic stability of the surf clams.

## Methods

### Collection and acclimatization of animals

Surf clams used in this study were obtained from the local Fish Market of Xiamen, Fujian Province, China, and maintained at 18 °C - 22 °C in tanks with recirculating seawater for a 2 week prior to bacterial infection. Prior to the experiments, the clams were acclimatized to the aquarium conditions for 2 week. The clams were fed twice daily with the marine algae, *Tetraselmis suecica* and *Isochrysis galbana*.

### Pathogenic challenge and survival rate

The clams were injected in the muscles with 100 μl of either *V*. *alginolyticus* (1 × 10^7^ CFU/ml) or *V. parahaemolyticus* (1 × 10^7^ CFU/ml) (These two *Vibrio* strains were graciously provided by Prof. Hu at South China Sea Institute of Oceanology, Chinese Academy of Sciences). Clams injected with 100 μl of phosphate buffered saline (PBS) were taken as controls [46]. Then the clams were returned to the tanks and maintained 18 °C - 22 °C until sampling. To measure the rate of survival of the clams, they were taken out of the tanks at definite time intervals of 24 h, 48 h, 60 h and 72 h post-infection and were examined for the presence of dead ones. The percentage of the surviving clams was calculated at each time interval for all the three groups.

### RNA isolation

Hemolymph was withdrawn from the adductor muscle of the control and the two *Vibrio* infected groups of clams 24 h post pathogenic injection, using a 0.5 mm diameter (25G) disposable needle. In total, 300 clams representing five biological replicates of bacterial challenge (Three treatments × five time points × five biological replicates × four clams per sample) were used for the expression analysis and stored at − 80 °C for RNA isolation. For RNA isolation, hemolymph was pooled from 20 individuals from each group. The experiment was repeated in triplicates. Briefly, the hemolymph was centrifuged at 3000 g for 10 min at 4 °C. The pellet was suspended in 250 μl of TRIzol (Invitrogen). Total RNA was isolated from each sample following the manufacturer’s protocols; followed by RNA purification with the RNeasy Mini kit (Qiagen) after treating with RNAase-free DNase I (NEB). The concentration and purity of isolated RNA was measured at 260 nm/280 nm (A260/A280) using spectrophotometer (Thermo Scientific). The RNA integrity was examined on Agilent 2100 Bioanalyser RNA Nano Chip (Agilent Technologies, USA) and by running on agarose gel electrophoresis.

### Illumina RNA-seq library preparation

To characterize the immune response among *V. parahaemolyticus-*infected groups of clams and to compare it with the control and *V*. *alginolyticus*-infected group, quantitative gene expression analysis for all the three groups of clams was performed using the Illumina RNA-seq technology on HiSeq 2000 platform [47, 48].

The extracted mRNA was converted to cDNA using SuperScript II reverse transcriptase (Invitrogen). cDNA was ligated to Illumina TruSeq RNA multiplex adaptor sequences using the TruSeq RNA sample prep kit (Illumina). Size-selected cDNA fragments of 200 bp (±25 bp) excised from a 2% agarose gel were amplified using Illumina PCR primers for paired-end reads (Illumina), and 15 cycles of the PCR programme comprising 98 °C for 30s, 98 °C for 10s, 65 °C for 30s and 72 °C for 30s, followed by an extension step of 5 min at 72 °C. After quantified by TBS380, two RNAseq libraries were sequenced in single lane on an Illumina Hiseq Xten sequencer (Illumina, San Diego, CA) for 2 × 150 bp paired-end reads.

### Data processing

Quality of reads was visualized with FastQC (http://www.bioinformatics.bbsrc.ac.uk/projects/fastqc). Initial removal of low-quality reads and multiplex index adaptor sequences (Illumina) was performed with edgeR [49], setting the quality threshold to minimum Phred score of 20/30. The rRNA sequence contamination, empty reads and sequences with copy number 1 were also removed.

The clean reads with quality scores greater than 20/30 (Q_20/30_) were assembled and mapped to the reference transcriptome of surf clam using edgeR; allowing only 1 or 2 bp mismatch [6]. For functional annotation, the differentially expressed gene (DEG) that mapped to only one gene in the reference database were taken and normalized to RPKM (Reads Per Kb per Million reads). The high quality short reads were submitted at the National Centre for Biological Information (NCBI) Short Read Archive (SRA; http://www.ncbi.nlm.nih.gov/sra/) under the accession numbers PRJNA560440.

### Gene ontology (GO) analyses

For GO analysis, the DEGs obtained from Illumina platform were mapped to terms in GO database version GO slim (http://www.geneontology.org) using Amigo (http://amigo.geneontology.org/) software. GO based functional analysis gave us a broad overview of the ontology content with respect to biological processes, cellular components and molecular functions [6, 9, 10, 47]. Default parameters were used to execute the analysis and the biological process ontology level 2 was selected [2, 13]. The up- and down-regulated genes from the annotated genes in GO database were identified using DAVID [47].

### Quantitative real time PCR (qRT-PCR)

cDNA was synthesized from 1 μg RNA using PrimeScript™ RT Reagent Kit Ver.2.0 (TaKaRa, Japan). The qRT-PCR reactions were performed in triplicate on a Light Cycler 480 platform (Roche) in a volume of 20 μl using 1 μL of diluted cDNA (1:10), 0.4 μL of each of the forward and reverse primers (10 mM) and 10 mL of 2× Master Mix (Roche, USA) with the cycling conditions as follows: 95 °C for 5 min, followed by 40 cycles of 94 °C for 5 s, 60 °C for 31 s [6, 50]. Beta actin was used as an internal control. The list of the genes chosen and the primers sequences for qRT-PCR are provided in Table 3.

### Measurement of bacterial count

The clams were injected with 5 mg/kg and 20 mg/kg of methotrexate (Sigma) and ruxolitinib (Sigma) respectively in their adductor muscles; followed by infection with 100 μl of 1 × 10^7^ CFU/ml of either *V. alginolyticus* or *V. parahaemolyticus*. Clams treated with dimethyl sulfoxide (DMSO) were used as control. Then the clams were returned to the tanks and maintained at 25 °C until sampling. The hemolymph was withdrawn from their adductor muscles after 24 h and plated onto TCBS media and incubated for 12 h at 37 °C and were observed for the growth of *Vibrio spp*. The positive colonies formed on the plates were counted and compared with control.

### Measurement of apoptosis index

For apoptotic index, the percentage of apoptotic hemocytes were analyzed in a flow cytometry (BD FACS Calibur) using fluorescence intensity based (photometric) assay targeting the hallmark of apoptosis (TUNEL (Terminal Deoxynucleotide Transferase dUTP Nick End Labeling) assay for detection of DNA fragmentation, the Annexin V assay for surface phosphatidylserine (PS) exposure, and fluorogenic caspase substrates to detect caspase activation) [50]. Approximately 10^5^ hemocytes were collected for apoptotic analysis, and total of 10,000 cells were obtained to detect in the flow cytometry. Finally, the result was conducted by FlowJo software.

### dsRNA-mediated gene silencing

dsRNA synthesis was performed using the MEGAscript T7 Transcription Kit (Life Technologies, USA) following the protocol. 50 μg of dsRNA was microinjected into the adductor muscles of the oyster to knockdown the expression of target genes. Then the clams were infected with *Vibrio spp*. as described before. Apoptosis indices and survival rate of the clams were measured. The relative expression of inflammatory molecules including glutathione-S-transferase (GST), apoptosis inhibitors (IAP) and tumor necrosis factor (TNF) were measured using qRT-PCR of mRNA isolated from these clams. The primers used to synthesize dsRNA are given in Table 3. Green fluorescent protein (GFP) was used as the internal control.

### Statistical analysis

One way ANOVA was used to analyze the mean differences among samples. Pair-wise t-test was then applied to compare gene expression levels between the two surf clam populations. All statistical analyses (avo functions and pairwise t-test functions)) were conducted in the R computation environment (http://www.r-project.org) [47]. *P*-values≤0.05 was considered statistically significant. The correlation statistics was performed and Pearson correlation coefficient (R) was calculated at *P* value< 0.0001.

## Data Availability

The high quality short reads were submitted at the National Centre for Biological Information (NCBI) Short Read Archive (SRA; http://www.ncbi.nlm.nih.gov/sra/) under the accession numbers PRJNA560440. Software input and output files generated in study is available from corresponding author.

## Abbreviations

AMPs: Antimicrobial peptides
DEGs: Differentially expressed genes
FDR: False discovery rate
GFP: Green fluorescent protein
GO: Gene ontology
GST: Glutathione-S-transferase
IAP: Apoptosis inhibitors
NLR: NACHT-leucine-rich repeat receptor
PAMPs: Pathogen-associated molecular patterns
PIAS: Protein inhibitors of activated STATs
PRRs: Pattern recognition receptors
PTPs: Protein tyrosine phosphatases
RLRs: Retinoic acid-inducible gene I -like receptors
RPKM: Reads Per Kb per Million reads
SOCS: Suppressors of cytokine signaling
STAT: Signal transducers and activators of transcription
TLR: Toll-like receptors
TNF: Tumor necrosis factor
TUNEL: Terminal Deoxynucleotide Transferase dUTP Nick End Labeling
VA: *Vibrio alginolyticus*
VP: *Vibrio parahaemolyticus*
